# Surgically induced scleral necrosis associated with concomitant tuberculosis infection: a diagnostic challenge

**DOI:** 10.3205/oc000212

**Published:** 2023-01-30

**Authors:** Raul E. Ruiz-Lozano, Alejandro Rodriguez-Garcia, Maria F. Colorado-Zavala, Carlos Alvarez-Guzman

**Affiliations:** 1 Tecnologico de Monterrey, School of Medicine and Health Sciences, Institute of Ophthalmology and Visual Sciences, Monterrey, Mexico

**Keywords:** antitubercular therapy, infectious scleritis, necrotizing scleritis, ocular tuberculosis, pterygium surgery, scleral inflammation, surgically induced scleral necrosis

## Abstract

**Objective:**

Surgically induced scleral necrosis (SISN) is a potentially blinding sequela that may occur after any ocular procedure. SISN in the context of active tuberculosis is seldom seen. We report a case of a patient with asymptomatic tuberculosis who developed SISN after pterygium surgery.

**Methods:**

A 76-year-old Mexican-mestizo woman from Veracruz, Mexico, was referred to our clinic because of severe disabling pain and scleral thinning in her right eye.

**Results:**

Tubercular-related SISN was finally diagnosed and managed successfully with antitubercular therapy, topical and systemic corticosteroids

**Conclusion:**

Tuberculosis must be considered as a differential diagnosis of high-risk patients in the context of refractory SISN in endemic countries.

## Introduction

Surgically induced scleral necrosis (SISN) is a serious complication that can occur after any ocular procedure. The onset time ranges from one day to more than 50 years after surgery. The most frequent SISN associated surgeries are pterygium excision and cataract extraction [1], [2]. While the former is mostly related to an infectious etiology, the latter is more probably associated with an underlying autoimmune disease [3]. The most common isolated pathogens of infectious SISN are bacteria, followed by fungus. Mycobacteria is an infrequent cause of SISN [4]. We report a case of a 76-year-old woman with tuberculosis (TB) infection who developed unilateral scleral necrosis 12 years after pterygium surgery.

## Case description

**Figure 1:**
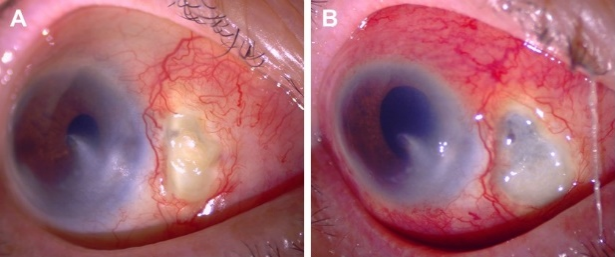
(A) At presentation. Anterior segment photograph of the right eye (RE) showing nasal marked ciliary injection, ischemic scleral necrosis and a corneal leukoma at the pterygium excision site. (B) At one month. Clinical photograph depicts progression of scleral necrosis, increased ciliary injection, and inflammatory activity was observed at the nasal peripheral cornea.

**Figure 2:**
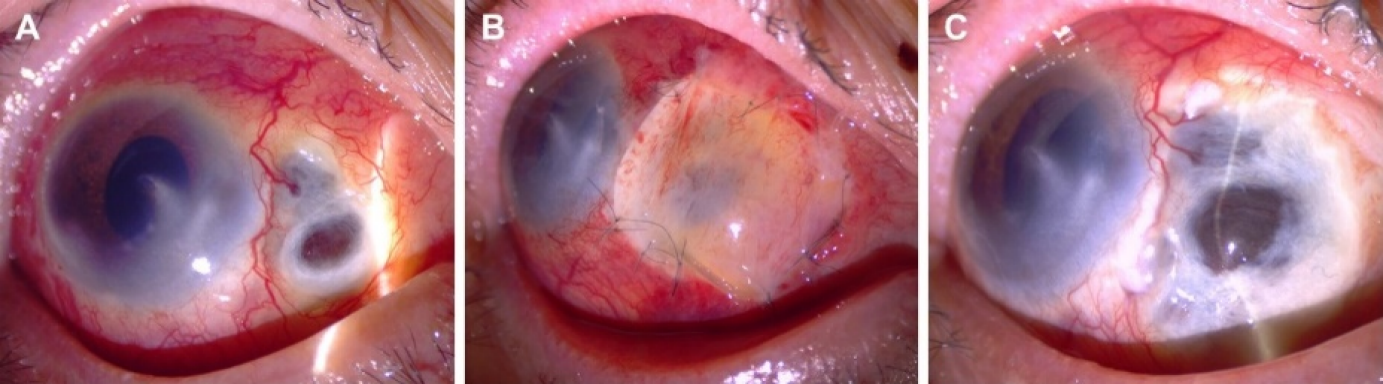
(A) At two months. Progression of necrosis with uveal exposure. (B) Area of necrosis with a corneal tectonic graft with conjunctival autograft. (C) One month after surgery the patch completely lysed.

**Figure 3:**
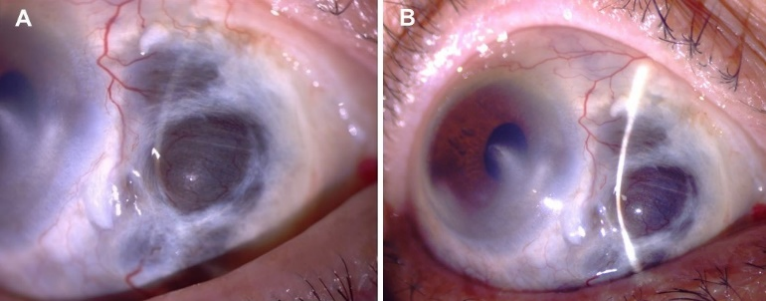
(A) After five and (B) seven months with antitubercular therapy, despite severe scleral thinning with uveal exposure, the eye remains quiet with no inflammation.

A 76-year-old woman from Veracruz, Mexico, was referred to the ophthalmology clinic for progressive pain and scleral thinning in the right eye (RE). Two months before, an episode of diffuse anterior scleritis was successfully managed with non-steroidal anti-inflammatory drugs (NSAIDs). The patient had bare-sclera pterygium surgery with adjunctive beta-irradiation therapy twelve years ago in the same eye. The past medical history was remarkable for uncontrolled diabetes mellitus (DM; HbA1c=14%). The review of systems was negative for fever, weight loss, and chronic cough. The best-corrected visual acuity was counting fingers at 3 feet in the RE and 20/25 in the left eye. Slit-lamp evaluation of the RE revealed marked ciliary injection, a nasal corneal leucoma, and scleral necrosis at the pterygium excision site (Figure 1A (Fig. 1)). Fundus examination of both eyes showed diffusely scattered microaneurysms and haemorrhages corresponding to a moderate non-proliferative diabetic retinopathy. The fellow eye examination was unremarkable. A work-up was negative for RF, anti-CCP, ANAs, ANCAs against PR3 and MPO. The infectious test included a negative FTA-Abs and a PPD test (8 mm induration; see [4] for a detailed classification of tuberculin skin reactions) [5]. Of notice, the patient had the BCG vaccination scar in her right arm. Topical treatment included loteprednol etabonate 0.5% every three hours, besifloxacin 0.6% QID, and aggressive lubrication. Systemic anti-inflammatory therapy consisted of 60 mg of oral prednisone daily, and 15 mg of weekly subcutaneous methotrexate (MTX) combined with an insulin regimen. After one month of therapy, the scleral necrosis progressed, and inflammatory activity was observed at the nasal peripheral cornea (Figure 1B (Fig. 1)). At this point, refractory SISN with peripheral corneal involvement was suspected, and thus, subcutaneous MTX was increased to 25 mg/week, and 1 g of intravenous methylprednisolone was prescribed for three days (Figure 1B (Fig. 1)). Moreover, empirical management with natamycin 5% QID and oral valaciclovir (1 g TID) for fungal and herpetic coverage was initiated. Unfortunately, the scleral necrosis continued progressing (Figure 2A (Fig. 2)); thus, a scleral biopsy, debridement, and a corneal tectonic graft with conjunctival autograft were performed due to impending corneal perforation (Figure 2B (Fig. 2)). Because of severe scleral thinning and a high risk of perforation, tissue sample was insufficient for culture analysis. Smear results, however, came out negative for bacteria, fungi, and mycobacteria. Polymerase chain reaction (PCR) analysis could not be performed due to a lack of insurance coverage. Pathology and smear results of the scarce necrotic tissue came out negative for bacteria, fungi, and mycobacteria. Because of a high risk of perforation, no other tissue was available for histopathologic study. One month later, the corneal tectonic graft lysed completely (Figure 2C (Fig. 2)). At this point, anti-tumor necrosis factor (anti-TNF)-α biologic therapy was considered; hence, an interferon-gamma release assay (IGRA) was ordered, resulting positive [Quanti-FERON^®^-TB Gold In-Tube (QFT-GIT) TB1 antigen=1.11 UI/ml]. The chest X-ray, CT-scan, and bronchoalveolar lavage were negative for tubercular disease. Assuming a relationship of the scleral necrosis with active TB infection, antitubercular therapy (ATT) was initiated with a regimen of isoniazid 300 mg, pyrazinamide 1500 mg, ethambutol 1200 mg, and rifampicin 600 mg [5]. The patient continued with topical steroids and antibiotics and a slow taper of oral prednisone for the next two months. The scleral necrosis subsided after one month of treatment. Her fasting glucose level was 117 mg/dl with a regimen of 850 mg daily metformin and 35 U of glargine insulin. An intensive 6-month regimen of ATT with four drugs was planned to reduce isoniazid and rifampicin for the next 6 months. The patient is currently in her seventh month of treatment and an excellent therapeutic response with no pain and no further scleral inflammation or necrosis are observed (Figure 3 (Fig. 3)).

## Discussion

SISN is a rare complication of any ocular surgery, being pterygium excision the most frequent one. In the most extensive SISN case series, O’Donoghue et al. reported a 70% female prevalence with a mean age of 68.2 years [6]. Furthermore, a large case series reported that SISN occurred most frequently after pterygium excision (63.4%), followed by cataract/lens extraction with 17.5%, and scleral buckle surgery at 11.3% [4]. Despite its elusive mechanism, the SISN hallmark is delayed wound healing. A myriad of pathogenic mechanisms such as excessive scleral cauterization, a bare sclera technique, and the use of adjunctive therapies like beta-irradiation and antimetabolites (mitomycin C and 5-fluorouracil) are related to SISN development after pterygium surgery [4]. Clinically SISN is characterized by severe pain and areas of capillary obstruction that give the sclera a “porcelainized” appearance or a violaceous discoloration secondary to thinning and uveal exposure (Figure 1B (Fig. 1)) [7], [8]. SISN related to pterygium surgery may be accompanied by an infectious process, in the setting of an autoimmune disease, or both. Distinguishing between postoperative infectious scleritis, which may lead to scleral necrosis, and SISN with concomitant scleral infection is challenging. Several factors predispose to concomitant infection in eyes with SISN, including vascular and local tissue disruption, cell apoptosis, and delayed wound healing [9]. On the other hand, postoperative infectious scleritis is another entity in which an interaction between a compromised immunologic response immediately after surgery and an infective microorganism occurs [4]. 

The high prevalence of DM in low-income countries like Mexico represents a health threat associated with developing opportunistic infections, including TB. The coexistence of both diseases represents a therapeutic challenge due to the high risk of failure with conventional treatment [10]. 

The current prevalence of latent TB infection (LTBI) in Mexico is unknown. In 2016, the national incidence of TB was estimated to be 24.7 per 100,000 inhabitants. However, this rate varies among different states [11], [12]. A systematic review demonstrated a three-fold increased risk of TB in people with DM than those without it [13]. Due to the association between both diseases in Mexico, the International Diabetes Federation (IDF) concluded that over 10% of TB cases could be attributed to DM [10]. Moreover, DM worsens the clinical outcome of TB and increases the risk for ocular TB [10]. 

The most common form of ocular TB results from hematogenous spread from distant foci of infection, including the lung and gastrointestinal tract [14]. In the eye, TB mostly presents as a choroidal granuloma. Such predilection is presumed to be related to the high oxygen tension present in the choroid [15]. Following posterior uveitis, granulomatous panuveitis is the second most common clinical presentation of ocular TB. Although TB is a common cause of scleritis, SISN associated with TB is rare [16]. Tuberculous scleritis may occur either as an immune-mediated reaction of circulating mycobacterial antigens or direct invasion of the sclera by *M. tuberculosis* [15]. TB scleritis usually presents as an anterior and necrotizing scleritis related to scleral ulceration [17].

The association between SISN and active TB is infrequent [16], [18], [19], [20], [21]. Notwithstanding, pterygium surgery is mostly related to infectious postoperative scleral necrosis [22], when concomitant TB infection, SISN development is more common after cataract/lens extraction [16], [20], [21], [23]. A single case has been reported in the medical literature in which SISN with active TB occurred 40 years after pterygium excision [18]. Table 1 (Tab. 1) shows the reported SISN cases with concomitant TB. Age at presentation ranged from 43 to 78 years old. Four cases had a prior history of cataract extraction with intraocular lens implantation [16], [20], [21], [23], one of periocular steroid injection [19], and one case had a history of pterygium excision with bare sclera technique 40 years prior [18]. Pain, redness, and blurred vision were the most frequent presenting symptoms. In two cases, a prior history of DM was recorded, one in a patient from Mexico [20]. Regarding treatment, patients who were managed with ATT, as in our case, had disease stabilization with no further recurrences [16], [20], [21], [23]. In one case, despite extensive diagnostic workup, including TB, the diagnosis was not made on time, and the globe was enucleated. Further histopathologic analysis revealed TB [19].

The combination of inadequate diagnostic criteria and the myriad of clinical symptoms of ocular TB mimicking other eye diseases frequently results in misdiagnosis [16]. In our patient, the diagnosis of TB was challenging. Neither a history of TB contact, common TB symptoms, radiologic findings, nor a positive PPD test were identified at the time of presentation. Moreover, the patient had a BCG vaccine. Disease progression despite aggressive medical and surgical therapy motivated consideration for biologic anti-TNF-α therapy. Before anti-TNF-α therapy, however, TB must be ruled out with both a negative IGRA and PPD [5]. In our case, the patient had a negative PPD and a positive IGRA. A positive IGRA result indicates infection but does not distinguish between active or latent disease. The latter occurs since IGRAs measure serum gamma-interferon correspondent to a cellular immune response to *M. tuberculosis*-specific antigens [24]. The National Institute for Health and Clinical Excellence (NICE) guidelines recommend a single-step IGRA test for TB screening, which yields a 70% sensitivity and 86% specificity for identifying LTBI progressing to active disease in two years and is more cost-effective than the two-step strategy (positive PPD with confirmatory IGRA) [24]. 

A meta-analysis by Metcalfe et al. concluded that IGRAs should not be used as a diagnostic tool for TB where the microbiological examination is available [25]. Microbiology tests yield low sensitivity in extrapulmonary TB, including the eye; thus, extrapulmonary TB lacks an objective gold-standard [26]. Moreover, tissue availability in ocular TB is not always feasible [27]. Therefore, clinical diagnosis of ocular TB is supported with a positive PPD, IGRA, or PCR analysis detecting mycobacterial DNA in ocular fluids or small samples [26], [27]. In our patient, the presence of a positive BCG vaccine mark and living in an endemic country raises suspicion of the negative PPD test. Cutaneous anergy, the presence of viral illnesses, and recent or very old TB episodes may result in a false negative PPD test [5]. In this case, however, the latter is unclear. Since ocular TB is considered a reactivation of dormant mycobacteria, patients with LTBI do not exhibit active infection manifestations [27], [28]. Nevertheless, since our patient had scleral inflammation, a positive IGRA, and responded favourably to the ATT, she may be considered as a manifest ocular TB (presumed) case [27].

## Conclusion

To our knowledge, this is only the second reported case of SISN occurring in the context of active TB long-term after pterygium surgery. Although rare, TB must always be considered in the differential diagnosis of refractory SISN, especially in endemic countries such as Mexico. Despite an initial negative PPD test, an IGRA is necessary when there is clinical suspicion of ocular TB. Moreover, in a patient with a high suspicion of autoimmune necrotizing scleritis and a history of previous ocular surgery, no matter the time elapsed from the surgical procedure, a scrape and culture of a scleral sample, along with PCR analysis for infectious agents, should be mandatory.

## Notes

### Authors’ ORCIDs

- Raul E. Ruiz Lozano: 0000-0001-7022-2395
- Alejandro Rodriguez-Garcia: 0000-0002-1419-2109
- Maria F. Colorado-Zavala: 0000-0001-5365-8461
- Carlos Alvarez-Guzman: 0000-0001-7236-950X

### Competing interests

The authors declare that they have no competing interests.

### Informed consent

Written informed consent for the publication of this case report and of the images was obtained from the patient.

## Figures and Tables

**Table 1:**
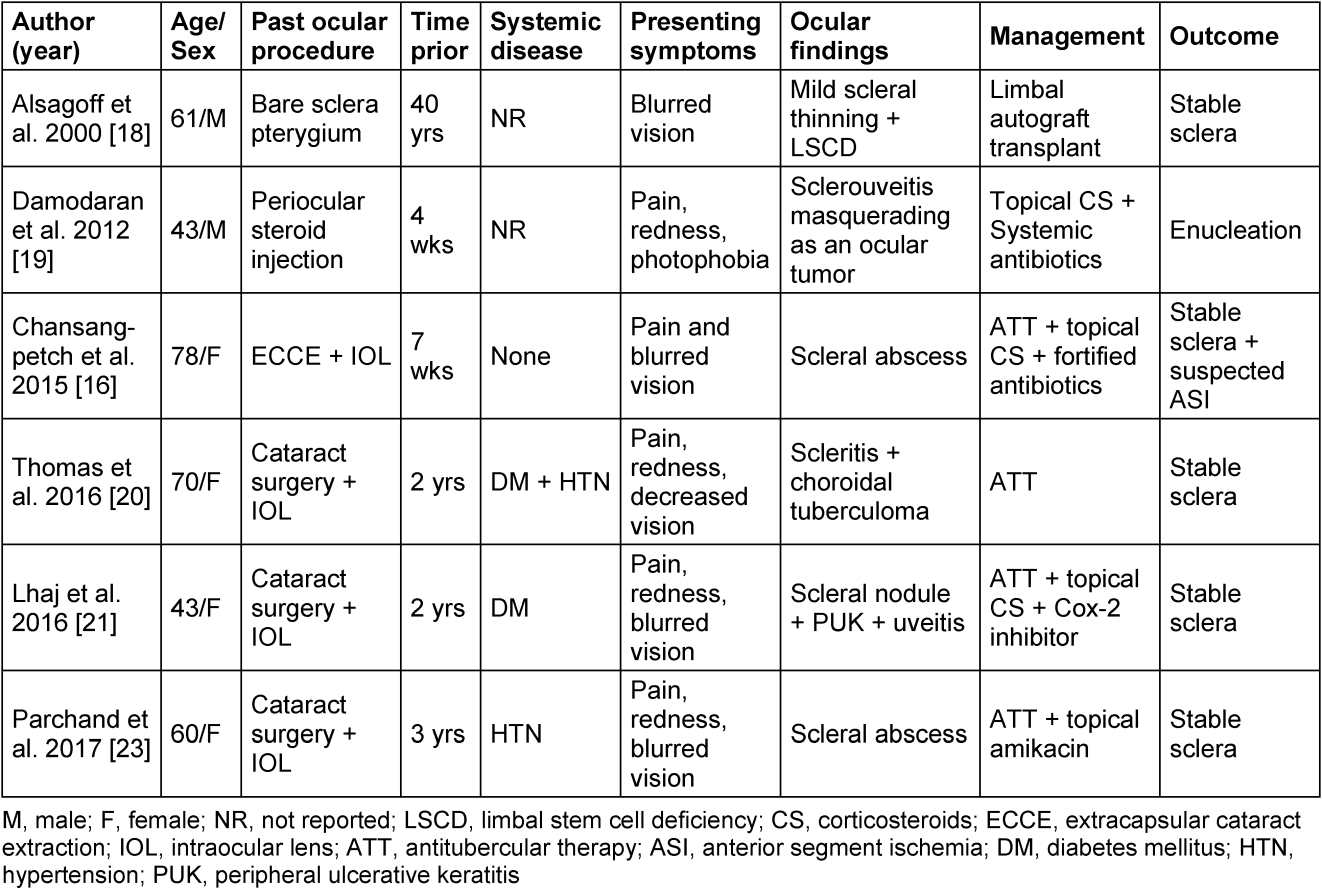
Reported cases of surgically induced scleral necrosis with concomitant active tuberculosis
